# Pretreatment with antibiotics is associated with reduced therapeutic response to atezolizumab plus bevacizumab in patients with hepatocellular carcinoma

**DOI:** 10.1371/journal.pone.0281459

**Published:** 2023-02-07

**Authors:** Kazuki Maesaka, Ryotaro Sakamori, Ryoko Yamada, Akira Doi, Yuki Tahata, Kazuyoshi Ohkawa, Masahide Oshita, Masanori Miyazaki, Takayuki Yakushijin, Yasutoshi Nozaki, Kengo Matsumoto, Satoshi Tanaka, Akira Kaneko, Sadaharu Iio, Takatoshi Nawa, Yukinori Yamada, Naoki Morishita, Takeo Usui, Naoki Hiramatsu, Yoshinori Doi, Mitsuru Sakakibara, Kazuho Imanaka, Yuichi Yoshida, Takahiro Kodama, Hayato Hikita, Tomohide Tatsumi, Tetsuo Takehara

**Affiliations:** 1 Department of Gastroenterology and Hepatology, Osaka University Graduate School of Medicine, Suita, Osaka, Japan; 2 Department of Hepatobiliary and Pancreatic Oncology, Osaka International Cancer Institute, Osaka, Osaka, Japan; 3 Department of Gastroenterology and Hepatology, Ikeda Municipal Hospital, Ikeda, Osaka, Japan; 4 Department of Gastroenterology and Hepatology, Osaka Police Hospital, Osaka, Osaka, Japan; 5 Department of Gastroenterology and Hepatology, Osaka General Medical Center, Osaka, Osaka, Japan; 6 Department of Gastroenterology and Hepatology, Kansai Rosai Hospital, Amagasaki, Hyogo, Japan; 7 Department of Gastroenterology and Hepatology, Toyonaka Municipal Hospital, Toyonaka, Osaka, Japan; 8 Department of Gastroenterology and Hepatology, National Hospital Organization Osaka National Hospital, Osaka, Osaka, Japan; 9 Department of Gastroenterology and Hepatology, Japan Community Healthcare Organization, Osaka Hospital, Osaka, Osaka, Japan; 10 Department of Gastroenterology and Hepatology, Hyogo Prefectural Nishinomiya Hospital, Nishinomiya, Hyogo, Japan; 11 Department of Gastroenterology and Hepatology, Higashiosaka City Medical Center, Higashiosaka, Osaka, Japan; 12 Department of Gastroenterology and Hepatology, Kaizuka City Hospital, Kaizuka, Osaka, Japan; 13 Department of Gastroenterology and Hepatology, Minoh City Hospital, Minoh, Osaka, Japan; 14 Department of Gastroenterology and Hepatology, Ashiya Municipal Hospital, Ashiya, Hyogo, Japan; 15 Department of Gastroenterology and Hepatology, Osaka Rosai Hospital, Sakai, Osaka, Japan; 16 Department of Gastroenterology and Hepatology, Otemae Hospital, Osaka, Osaka, Japan; 17 Department of Gastroenterology and Hepatology, Yao Municipal Hospital, Yao, Osaka, Japan; 18 Department of Gastroenterology and Hepatology, Itami City Hospital, Itami, Hyogo, Japan; 19 Department of Gastroenterology and Hepatology, Suita Municipal Hospital, Suita, Osaka, Japan; Al-Azhar University, EGYPT

## Abstract

**Aim:**

Alterations in microbial composition of gut microbiota due to antibiotics (ATB) may lead to resistance to immune checkpoint inhibitors (ICIs). This study aimed to assess the impact of ATB use on therapeutic response in patients with hepatocellular carcinoma (HCC) receiving atezolizumab plus bevacizumab.

**Methods:**

This study retrospectively analyzed 105 patients with HCC treated with atezolizumab plus bevacizumab as a primary systemic therapy from prospectively-registered, multicenter, cohorts. Nineteen patients who received prior ATB were included in the ATB (+) group; 86 patients who did not receive prior ATB were included in the ATB (-) group. The therapeutic outcomes were compared between the two groups.

**Results:**

Most of the patients’ baseline characteristics were not significantly different between the two groups. The objective response rates according to the Response Evaluation Criteria in Solid Tumors version 1.1 (RECIST v1.1) (30.1% vs. 11.1%; *p* = 0.143) and modified RECIST (mRECIST) (44.6% vs. 27.8%; *p* = 0.190) were not significantly different between the ATB (-) and ATB (+) groups. The disease control rates were higher in the ATB (-) group than in the ATB (+) group according to RECIST v1.1 (74.7% vs. 44.4%; *p* = 0.012) and mRECIST (78.3% vs. 50.0%; *p* = 0.020). Prior ATB use was found to be independently associated with radiological progressive disease of the first therapeutic assessment. The median progression-free survival according to RECIST v1.1 (9.1 months vs. 3.0 months; *p* = 0.049) and mRECIST (9.1 months vs. 3.0 months; *p* = 0.036), and overall survival (not reached vs. 11.4 months; *p* = 0.015) were longer in the ATB (-) group than in the ATB (+) group.

**Conclusions:**

Prior ATB use was associated with reduced therapeutic responses in patients with HCC receiving atezolizumab plus bevacizumab.

## Introduction

Atezolizumab combined with bevacizumab therapy in patients with unresectable hepatocellular carcinoma (HCC) was reported to be superior to sorafenib therapy in terms of overall survival (OS) and progression-free survival (PFS) in the Imbrave150 trial [1, 2]. Atezolizumab plus bevacizumab is the primary systemic therapy option in patients with unresectable HCC according to clinical practice guidelines worldwide [3–6]. However, approximately 20−30% of patients treated with atezolizumab plus bevacizumab are non-responders [1, 2, 7–9].

Immune checkpoint inhibitor (ICI) responses have been attributed to several factors, such as tumor-infiltrating lymphocytes [10, 11], programmed cell death protein 1/programmed cell death protein ligand 1 expression [12, 13], tumor mutational burden [13, 14], tumor associated antigen expression [15], and gene expression profiles [16]. The impact of gut microbiota on ICI responses has been reported recently [17–21]. The diversity and composition of patient gut microbiomes may affect ICI responses in patients with some types of malignancies [22]. Gut microbiome with higher taxa richness and greater numbers of genes in patients with HCC was observed in ICI responders than in non-responders [23].

Antibiotics (ATB) affect the integrity of the gut microbiota and lead to the reduced diversity of the gut microbiota [24]. Previous studies have suggested that modulation of the gut microbiota by ATB use prior to or during ICI therapy may be associated with resistance to ICIs in patients with several types of malignancies [25–31]. However, studies investigating the association between ICI response and ATB use are lacking in patients with HCC.

This multicenter study including patients with HCC treated with atezolizumab plus bevacizumab as the primary systemic chemotherapy according to clinical practice guidelines was conducted to assess the impact of ATB use on the therapeutic responses in real-world settings.

## Methods

### Patients

Patients with HCC treated with atezolizumab plus bevacizumab as the primary systemic therapy were retrospectively analyzed using cohorts prospectively registered via the Osaka Liver Forum consisting of Osaka University Hospital and 18 affiliated hospitals. All study participants provided written informed consent prior to enrolment. This study was carried out in accordance with the ethical principles of the Declaration of Helsinki. The study design was approved by the Institutional Review Board of Osaka University Hospital, Osaka International Cancer Institute, Ikeda Municipal Hospital, Osaka Police Hospital, Osaka General Medical Center, Kansai Rosai Hospital, Toyonaka Municipal Hospital, National Hospital Organization Osaka National Hospital, Japan Community Healthcare Organization, Hyogo Prefectural Nishinomiya Hospital, Higashiosaka City Medical Center, Kaizuka City Hospital, Minoh City Hospital, Ashiya Municipal Hospital, Osaka Rosai Hospital, Otemae Hospital, Yao Municipal Hospital, Itami City Hospital, and Suita Municipal Hospital (UMIN Clinical Trials Registry: 000034611).

HCC was diagnosed with histological findings or radiological findings using modalities such as dynamic contrast-enhanced computed tomography (CT) and magnetic resonance imaging (MRI) scans based on the diagnostic criteria of the American Association for the Study of Liver Diseases [32, 33]. The inclusion criteria for the present study were as follows: (1) patients receiving atezolizumab plus bevacizumab as a primary systemic therapy between October 2020 and January 2022, (2) patients with unresectable HCC that was not amenable to locoregional therapy such as transarterial chemoembolization and radiofrequency ablation due to metastatic or local progression, and that was eligible for ICI therapy, (3) patients with an Eastern Cooperative Oncology Group performance status of 0 or 1, and (4) patients with Child-Pugh class A or B hepatic reserve. The exclusion criteria were as follows: (1) patients who did not undergo liver imaging using contrast media, (2) patients who had observation periods shorter than six weeks, and (3) patient who were participating in a clinical trial.

Eligible patients were classified into two groups based on the administration of any oral or intravenous ATB up to 30 days prior to the initiation of atezolizumab plus bevacizumab therapy. Patients who were administered ATB within 30 days after the atezolizumab plus bevacizumab initiation were excluded from the study to allow for the investigation of the association between ATB exposure only prior to atezolizumab plus bevacizumab and therapeutic response to the treatment in this study. This was because early ATB use after the atezolizumab plus bevacizumab initiation may be associated with adverse events (AEs) during the treatment, which would confound the treatment outcome data.

### Atezolizumab plus bevacizumab therapy

All patients were treated with intravenous atezolizumab (1,200 mg) plus bevacizumab (15 mg/kg) once every three weeks. If any unacceptable or serious AE related to either drug occurred, the administration was interrupted until the symptoms diminished to grade 1 or 2 based on the National Cancer Institute Common Terminology Criteria for Adverse Events, version 4.0. Subsequent treatment was determined by the attending physician when atezolizumab plus bevacizumab therapy was discontinued due to confirmed tumor progression or unacceptable AEs.

### Evaluation of therapeutic response and hepatic reserve

Using dynamic enhanced CT or MRI at each institution, the therapeutic responses were assessed every six to eight weeks after treatment initiation according to the Response Evaluation Criteria in Solid Tumors version 1.1 (RECIST v1.1) and the modified RECIST (mRECIST) [34, 35]. The objective response rate (ORR) was defined as the percentage of the sum of patients with complete response (CR) and partial response (PR). The disease control rate (DCR) was defined as the percentage of the sum of patients with CR, PR, or stable disease (SD). Based on the best objective responses, patients were assigned to the CR, PR, and SD categories. OS was defined as the time from the first day of treatment to the day of death or last follow-up. PFS was defined as the time from the first day of treatment to the day of progressive disease (PD) onset or death. The hepatic reserve was classified according to Child-Pugh classifications, albumin-bilirubin (ALBI) scores, and modified albumin-bilirubin (mALBI) grades [36, 37].

### Statistical analysis

Statistical analyses were performed using SPSS version 24.0 (IBM). Statistical significance was set at *p* < 0.05. Clinical parameter values are presented as percentages (categorical variables) and medians and ranges (continuous variables). Categorical variables were compared between the groups using the chi-square or Fisher’s exact tests. Continuous variables were compared between the groups using the Mann-Whitney U test. The OS and PFS were analyzed using the Kaplan-Meier method, and the differences of OS and PFS between the groups were analyzed using the log-rank test. Cox proportional hazards regression models were used to identify predictors associated with OS and PFS. Logistic regression analyses were performed to identify predictors associated with radiological PD of the first therapeutic assessment.

## Results

### Patient characteristics

**Fig 1:**
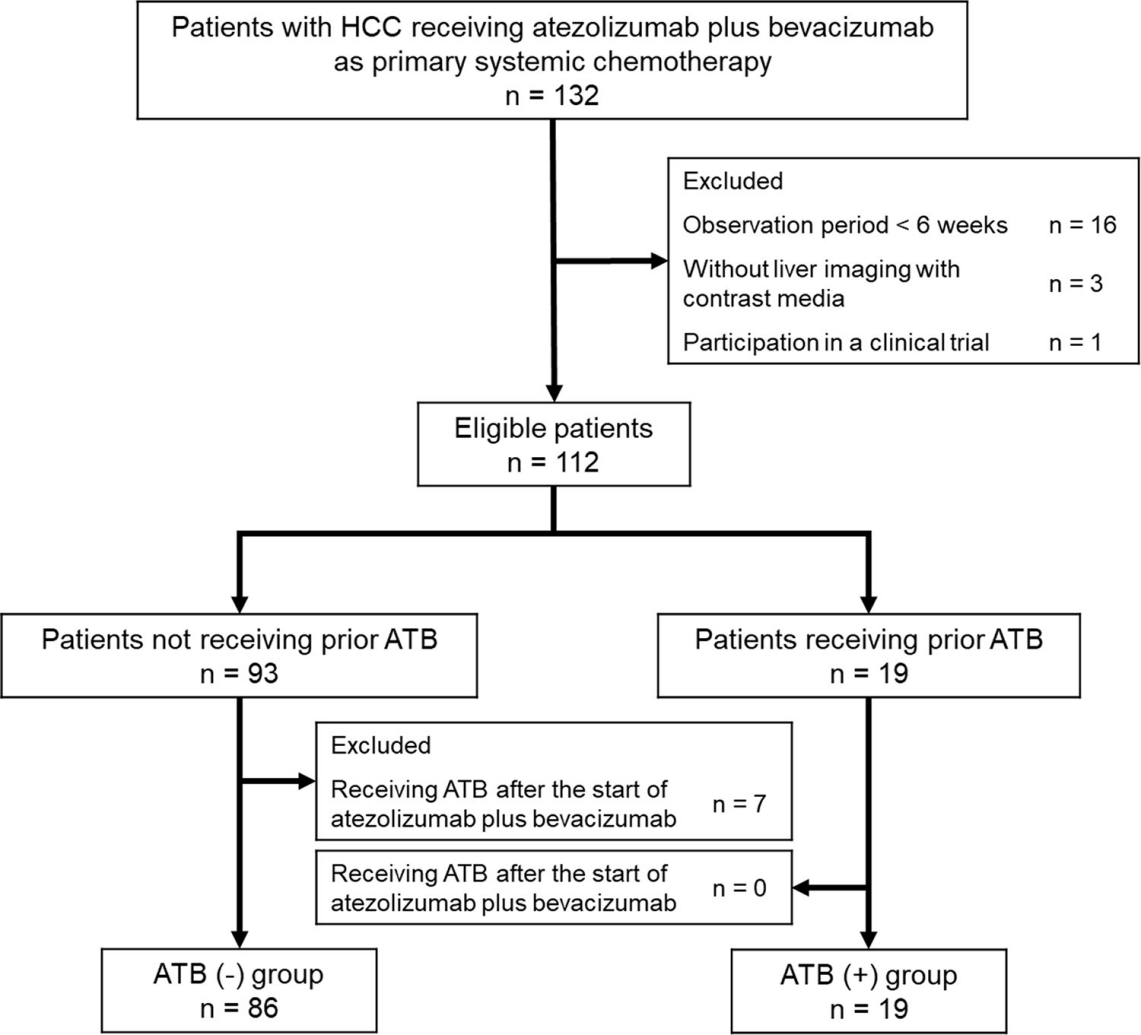
Flowchart of patient selection. Abbreviations: HCC, hepatocellular carcinoma; ATB, antibiotics.

A total of 132 patients with HCC treated with atezolizumab plus bevacizumab as the primary systemic therapy were initially registered for the present study (**Fig 1**). Of the 132 patients, 16 were excluded due to a short observation period, three were excluded due to missing contrast-enhanced liver imaging, and one was excluded due to participation in a clinical trial. A total of 19 patients received ATB prior to the initiation of atezolizumab plus bevacizumab (ATB (+) group) and 93 did not (ATB (-) group). However, seven patients in the ATB (-) group were excluded due to receiving ATB within 30 days after the atezolizumab plus bevacizumab initiation. Two patients in the ATB (+) group were continuously administered rifaximin prior to the initiation of the treatment and were not excluded from the analyses. Therefore, the final analysis included 86 patients in the ATB (-) group and 19 patients in the ATB (+) group.

Most of patients’ baseline characteristics were not significantly different between the two groups (**Table 1**). However, the ATB (+) group included younger patients, a greater percentage of female patients, patients with higher ALBI scores, and patients with larger intrahepatic tumors than the ATB (-) group. The median C-reactive protein (CRP) level was significantly higher in the ATB (+) group than that in the ATB (-) group (ATB (-): 0.20 mg/dL vs. ATB (+): 0.52 mg/dL; *p* = 0.002). Neither of the values were indicative of infection. In fact, the infection in the ATB (+) group was considered to be clinically cured at the initiation of atezolizumab plus bevacizumab. The median observation periods were 7.0 months (1.4–14.5 months) in the ATB (-) group and 5.1 months (1.4–14.0 months) in the ATB (+) group.

**Table 1:** Comparisons of the characteristics between the ATB (-) and ATB (+) groups.

Variable		ATB (-)	ATB (+)	*p* value
		n = 86	n = 19	
**Age, years**	**Median (range)**	**76 (49 to 93)**	**73 (54 to 89)**	**0.066**
**Sex, n (%)**	**Male**	**72 (83.7)**	**12 (63.2)**	**0.058**
	**Female**	**14 (16.3)**	**7 (36.8)**	
**ECOG PS, n (%)**	**0**	**81 (94.2)**	**18 (94.7)**	**1.000**
	**1**	**5 (5.8)**	**1 (5.3)**	
**Etiology, n (%)**	**Viral**	**42 (48.8)**	**8 (42.1)**	**0.595**
	**HBV/HCV/HBV+HCV**	**11 (12.8)/30 (34.9)/1 (1.2)**	**2 (10.5)/6 (31.6)/0 (0.0)**	
	**Non-viral**	**44 (51.2)**	**11 (57.9)**	
	**Alcohol/Others**	**24 (27.9)/20 (23.3)**	**5 (26.3)/6 (31.6)**	
**Child-Pugh score, n (%)**	**5**	**46 (53.5)**	**7 (36.8)**	**0.189**
	**6 or 7**	**40 (46.5)**	**12 (63.2)**	
**ALBI score**	**Median (range)**	**-2.36 (-3.43 to -1.49)**	**-2.02 (-3.28 to -1.34)**	**0.080**
**mALBI grade, n (%)**	**1 or 2a**	**51 (59.3)**	**9 (47.4)**	**0.341**
	**2b or 3**	**35 (40.7)**	**10 (52.6)**	
**Platelet count, x 10** ^**4**^ **/μL**	**Median (range)**	**13.9 (5.0 to 28.9)**	**15.5 (4.8 to 34.1)**	**0.236**
**Maximum intrahepatic tumor size, mm**	**Median (range)**	**24 (0 to 132)**	**36 (0 to 170)**	**0.082**
**Intrahepatic tumor number, n (%)**	**≤ 4**	**51 (59.3)**	**10 (52.6)**	**0.594**
	**≥ 5**	**35 (40.7)**	**9 (47.4)**	
**Macrovascular invasion, n (%)**	**Absent**	**72 (83.7)**	**13 (68.4)**	**0.192**
	**Present**	**14 (16.3)**	**6 (31.6)**	
**Extrahepatic metastasis, n (%)**	**Absent**	**56 (65.1)**	**10 (52.6)**	**0.308**
	**Present**	**30 (34.9)**	**9 (47.4)**	
**BCLC stage, n (%)**	**A or B**	**43 (50.0)**	**7 (36.8)**	**0.299**
	**C**	**43 (50.0)**	**12 (63.2)**	
**AFP, ng/mL**	**Median (range)**	**15 (1 to 496493)**	**24 (3 to 368469)**	**0.552**
**NLR**	**Median (range)**	**2.54 (0.76 to 7.50)**	**2.15 (0.77 to 4.90)**	**0.265**
**WBC, /μL**	**Median (range)**	**4350 (1900 to 9860)**	**4200 (2600 to 6680)**	**0.963**
**CRP, mg/dL**	**Median (range)**	**0.20 (0.01 to 9.05)**	**0.52 (0.03 to 2.83)**	**0.002**

Abbreviations: AFP, α-fetoprotein; ALBI, albumin-bilirubin; ATB, antibiotics; BCLC, Barcelona Clinic Liver Cancer; CRP, C-reactive protein; ECOG PS, Eastern Cooperative Oncology Group performance status; HBV, hepatitis B virus; HCV, hepatitis C virus; mALBI, modified albumin-bilirubin; NLR, neutrophil-to-lymphocyte ratio; WBC, white blood cell.

The ATB used by patients in the ATB (+) group included cefmetazole (n = 4), sulbactam/cefoperazone (n = 4), cefazolin (n = 2), cefepime (n = 1), cefpodoxime proxetil (n = 1), sulbactam/ampicillin (n = 1), tazobactam/piperacillin (n = 1), amoxicillin/clavulanate (n = 1), levofloxacin (n = 1), teicoplanin (n = 1), and rifaximin (n = 2). The indications for ATB use included as a treatment for cholangitis (n = 2), cholecystitis (n = 2), hepatic abscess (n = 2), pyothorax (n = 1), and cystitis (n = 1) and as prophylaxis after transarterial chemoembolization (n = 3), liver biopsy (n = 2), endoscopic ultrasound-guided fine needle aspiration (n = 2), endoscopic retrograde cholangiopancreatography (n = 1), hepatic arterial infusion chemotherapy (n = 1), and hepatic encephalopathy (n = 2). Details regarding antibiotic treatment and duration among patients in the ATB (+) group are shown in **S1 Table**.

### Comparisons of therapeutic response

The best therapeutic responses for CR, PR, SD, and PD according to mRECIST were observed in 8, 29, 28, and 18 patients in the ATB (-) group and in 0, 5, 4, and 9 patients in the ATB (+) group, respectively (**Table 2**). The ORRs measured using mRECIST were not significantly different between the groups (ATB (-): 44.6% vs. ATB (+): 27.8%; *p* = 0.190). The DCRs were significantly higher in the ATB (-) group than in the ATB (+) group (ATB (-): 78.3% vs. ATB (+): 50.0%; *p* = 0.020). Similarly, the best therapeutic responses for CR, PR, SD, and PD according to RECIST v1.1 were observed in 2, 23, 37, and 21 patients in the ATB (-) group and in 0, 2, 6, and 10 patients in the ATB (+) group, respectively (**Table 2**). The ORRs measured using RECIST v1.1 were not significantly different between the groups (ATB (-): 30.1% vs. ATB (+): 11.1%; *p* = 0.143). The DCRs were significantly higher in the ATB (-) group than in the ATB (+) group (ATB (-): 74.7% vs. ATB (+): 44.4%; *p* = 0.012). The rates of drug interruption before the first radiological assessment did not differ between the ATB (-) and ATB (+) groups (ATB (-): 14.0% vs. ATB (+): 21.1%; *p* = 0.483). In addition, the DCRs in 8 patients receiving prior ATB as treatment for infection were lower than those in 11 patients receiving prior ATB as prophylaxis (42.9% and 28.6% according to mRECIST and RECIST v1.1, respectively vs. 54.5% and 54.5% according to mRECIST and RECIST v1.1, respectively, not significant).

**Table 2:** Comparisons of therapeutic efficacy between the ATB (-) and ATB (+) groups.

Therapeutic efficacy		ATB (-)	ATB (+)	*p* value
		n = 86	n = 19	
**mRECIST**	**CR/PR/SD/PD/NE, n**	**8/29/28/18/3**	**0/5/4/9/1**	
	**ORR, n (%)**	**37 (44.6)**	**5 (27.8)**	**0.190**
	**DCR, n (%)**	**65 (78.3)**	**9 (50.0)**	**0.020**
**RECIST v1.1**	**CR/PR/SD/PD/NE, n**	**2/23/37/21/3**	**0/2/6/10/1**	
	**ORR, n (%)**	**25 (30.1)**	**2 (11.1)**	**0.143**
	**DCR, n (%)**	**62 (74.7)**	**8 (44.4)**	**0.012**

Abbreviations: ATB, antibiotics; CR, complete response; DCR, disease control rate; mRECIST, modified Response Evaluation Criteria in Solid Tumors; NE, not evaluated; ORR, objective response rate; PD, progressive disease; PR, partial response; RECIST v1.1, Response Evaluation Criteria in Solid Tumors version 1.1; SD, stable disease

The maximum intrahepatic tumor size, macrovascular invasion, serum α-fetoprotein (AFP) level, and prior ATB were identified as significant factors associated with PD according to mRECIST. Prior ATB was the only independent factor associated with PD according to mRECIST (**Table 3**). According to RECIST v1.1, the macrovascular invasion, neutrophil-to-lymphocyte ratio (NLR), and prior ATB were identified as significant factors associated with PD, and prior ATB was the only independent factor associated with PD (**Table 4**).

**Table 3:** Univariate and multivariate analyses of factors associated with progressive disease according to mRECIST.

Variable	Category	Univariate analysis	*p* value	Multivariate analysis	*p* value
		Odds ratio (95% CI)		Odds ratio (95% CI)	
**Age, years**	**≥ 75**	**0.553 (0.226–1.353)**	**0.195**		
**Sex**	**Female**	**1.224 (0.417–3.598)**	**0.713**		
**ECOG PS**	**1**	**2.958 (0.559–15.650)**	**0.202**		
**Etiology**	**Non-viral**	**0.879 (0.654–1.182)**	**0.394**		
**Child-Pugh score**	**6 or 7**	**0.929 (0.384–2.243)**	**0.869**		
**mALBI grade**	**2b or 3**	**1.219 (0.504–2.950)**	**0.661**		
**Platelet count, x 10** ^**4**^ **/μL**	**≤ 14.0**	**1.250 (0.516–3.030)**	**0.621**		
**Maximum intrahepatic tumor size, mm**	**≥ 50**	**2.682 (1.056–6.816)**	**0.038**	**1.721 (0.612–4.835)**	**0.303**
**Intrahepatic tumor number**	**≥ 5**	**1.940 (0.796–4.728)**	**0.145**		
**Macrovascular invasion**	**Present**	**3.765 (1.348–10.511)**	**0.011**	**2.516 (0.828–7.647)**	**0.104**
**Extrahepatic metastasis**	**Present**	**1.477 (0.602–3.621)**	**0.394**		
**BCLC stage**	**C**	**2.111 (0.841–5.302)**	**0.112**		
**AFP, ng/mL**	**≥ 400**	**3.429 (1.318–8.922)**	**0.012**	**2.774 (0.972–7.916)**	**0.057**
**NLR**	**≥ 3**	**2.316 (0.922–5.817)**	**0.074**		
**CRP, mg/dL**	**≥ 0.25**	**2.500 (0.991–6.307)**	**0.052**		
**ATB**	**With**	**3.611 (1.250–10.436)**	**0.018**	**3.161 (1.003–9.964)**	**0.049**

Abbreviations: AFP, α-fetoprotein; ATB, antibiotics; BCLC, Barcelona Clinic Liver Cancer; CI, confidence interval; CRP, C-reactive protein; ECOG PS, Eastern Cooperative Oncology Group performance status; mALBI, modified albumin-bilirubin; mRECIST, modified Response Evaluation Criteria in Solid Tumors; NLR, neutrophil-to-lymphocyte ratio.

**Table 4:** Univariate and multivariate analyses of factors associated with progressive disease according to RECIST v1.1.

Variable	Category	Univariate analysis	*p* value	Multivariate analysis	*p* value
		Odds ratio (95% CI)		Odds ratio (95% CI)	
**Age, years**	**≥ 75**	**0.474 (0.200–1.124)**	**0.090**		
**Sex**	**Female**	**0.960 (0.331–2.788)**	**0.940**		
**ECOG PS**	**1**	**2.393 (0.455–12.584)**	**0.303**		
**Etiology**	**Non-viral**	**0.885 (0.667–1.175)**	**0.398**		
**Child-Pugh score**	**6 or 7**	**1.129 (0.485–2.632)**	**0.778**		
**mALBI grade**	**2b or 3**	**1.821 (0.776–4.278)**	**0.169**		
**Platelet count, x 10** ^**4**^ **/μL**	**≤ 14.0**	**1.775 (0.750–4.202)**	**0.192**		
**Maximum intrahepatic tumor size, mm**	**≥ 50**	**1.969 (0.796–4.872)**	**0.143**		
**Intrahepatic tumor number**	**≥ 5**	**1.600 (0.683–3.749)**	**0.279**		
**Macrovascular invasion**	**Present**	**3.728 (1.350–10.291)**	**0.011**	**2.805 (0.960–8.195)**	**0.059**
**Extrahepatic metastasis**	**Present**	**1.578 (0.666–3.739)**	**0.300**		
**BCLC stage**	**C**	**2.355 (0.970–5.718)**	**0.059**		
**AFP, ng/mL**	**≥ 400**	**2.526 (0.997–6.404)**	**0.051**		
**NLR**	**≥ 3**	**2.567 (1.050–6.276)**	**0.039**	**2.259 (0.868–5.877)**	**0.095**
**CRP, mg/dL**	**≥ 0.25**	**2.303 (0.957–5.540)**	**0.062**		
**ATB**	**With**	**3.690 (1.287–10.580)**	**0.015**	**3.378 (1.119–10.193)**	**0.031**

Abbreviations: AFP, α-fetoprotein; ATB, antibiotics; BCLC, Barcelona Clinic Liver Cancer; CI, confidence interval; CRP, C-reactive protein; ECOG PS, Eastern Cooperative Oncology Group performance status; mALBI, modified albumin-bilirubin; NLR, neutrophil-to-lymphocyte ratio; RECIST v1.1, Response Evaluation Criteria in Solid Tumors version 1.1.

### Comparisons of PFS and OS

**Fig 2:**
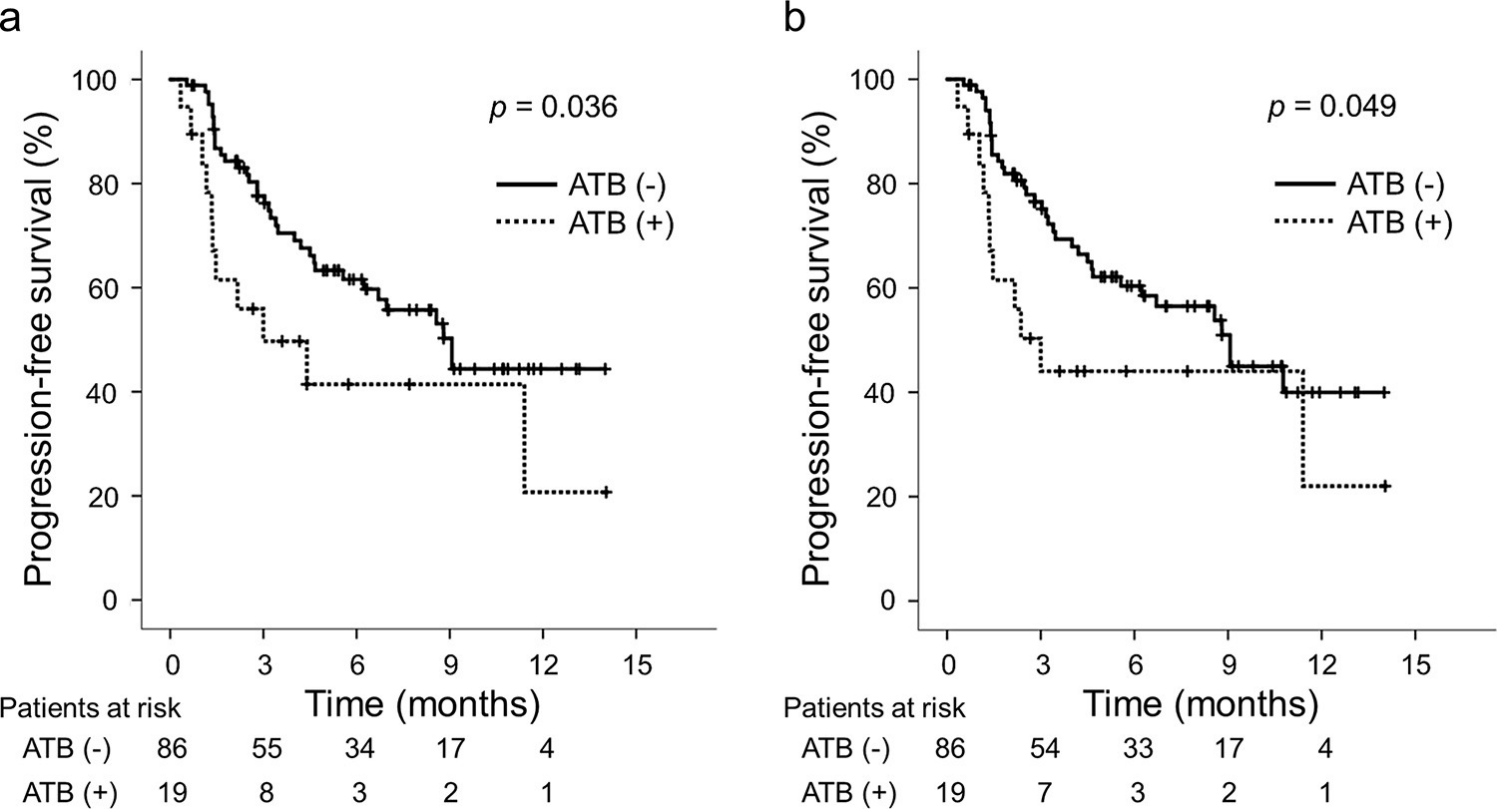
Comparisons of the ATB (+) and ATB (-) groups in terms of PFS (a) based on mRECIST and (b) based on RECIST v1.1. Abbreviations: ATB, antibiotics; RECIST v1.1, Response Evaluation Criteria in Solid Tumors version 1.1; mRECIST, modified RECIST; PFS, progression-free survival.

During the observation period, 33 (38.4%) and 34 (39.5%) patients in the ATB (-) group ultimately developed tumor progression based on mRECIST and RECIST v1.1, respectively, while 10 (52.6%) patients in the ATB (+) group finally developed tumor progression based on both mRECIST and RECIST v1.1. The median PFS was significantly longer in the ATB (-) group than in the ATB (+) group based on mRECIST (ATB (-): 9.1 months vs. ATB (+): 3.0 months; *p* = 0.036; **Fig 2A**) and based on RECIST v1.1 (ATB (-): 9.1 months vs. ATB (+): 3.0 months; *p* = 0.049; **Fig 2B**). The intrahepatic tumor number, macrovascular invasion, Barcelona Clinic Liver Cancer (BCLC) stage, NLR, CRP level, and prior ATB were identified as significant predictors associated with the PFS based on mRECIST. The intrahepatic tumor number and macrovascular invasion were identified as independent predictors associated with the PFS based on mRECIST (**S2 Table**). Similarly, the intrahepatic tumor number, macrovascular invasion, BCLC stage, NLR, and CRP level were identified as significant predictors associated with the PFS based on RECIST v1.1. The intrahepatic tumor number and macrovascular invasion were identified as independent predictors associated with the PFS based on RECIST v1.1 (**S3 Table**).

**Fig 3:**
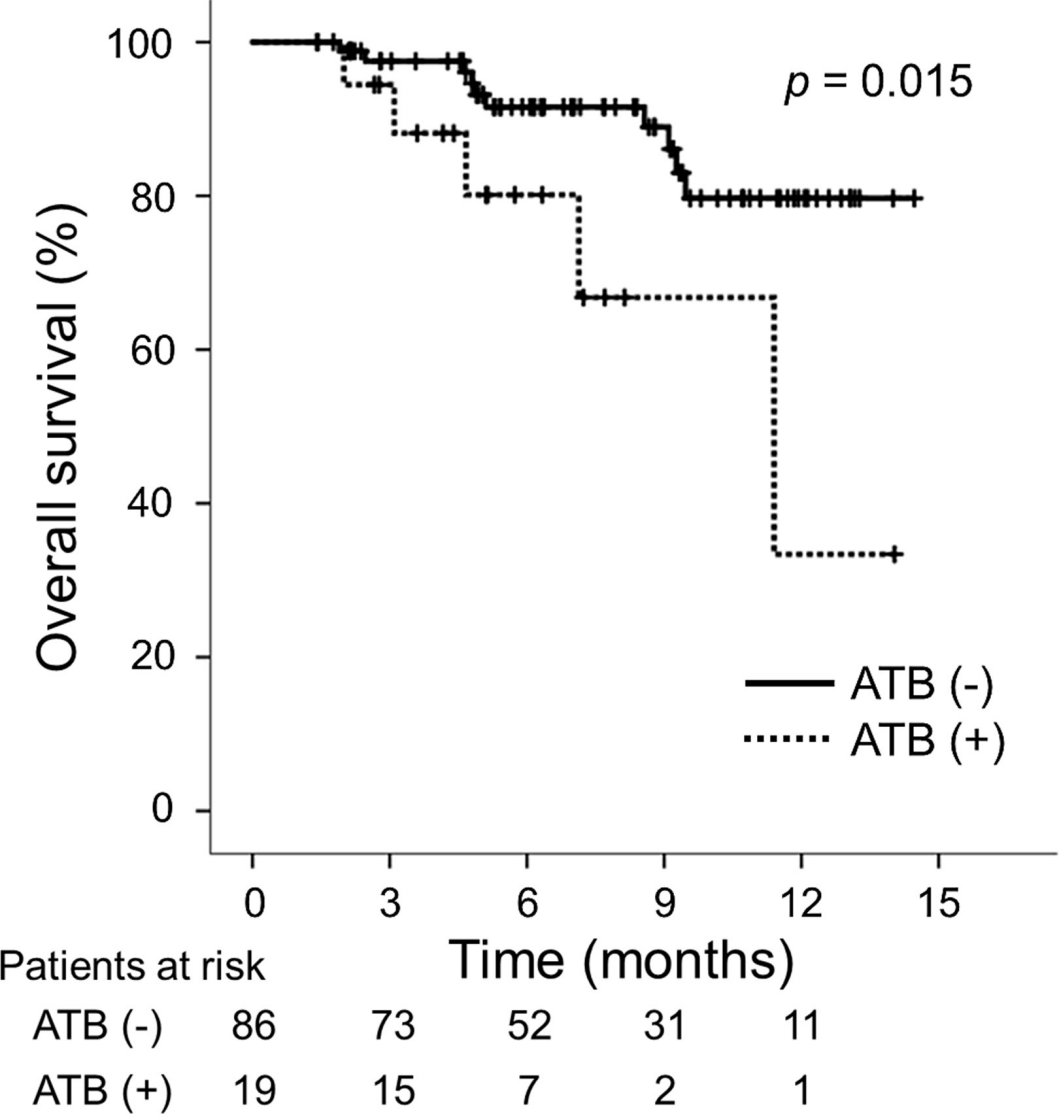
Comparison of the ATB (+) and ATB (-) groups in terms of OS. Abbreviations: ATB, antibiotics; OS, overall survival.

During the observation period, 10 (11.6%) patients in the ATB (-) group and 5 (26.3%) in the ATB (+) group died. The median OS was significantly longer in the ATB (-) group than in the ATB (+) group (ATB (-): not reached vs. ATB (+): 11.4 months; *p* = 0.015; **Fig 3**). The Child-Pugh score, mALBI grade, intrahepatic maximum tumor size and tumor number, macrovascular invasion, serum AFP level, NLR, CRP level, and prior ATB were identified as significant predictors associated with the OS, while the Child-Pugh score, intrahepatic tumor number, macrovascular invasion, serum AFP level, and NLR were identified as independent predictors associated with the OS (**S4 Table**).

### Comparisons of adverse events

The frequency of major AEs including any infection and any immune-related AE treated with steroids is shown in **Table 5**. There were no significant differences in terms of each AE and any AE of grade ≥ 3 between the groups.

**Table 5:** Comparisons of adverse events between the ATB (-) and ATB (+) groups.

Adverse events	ATB (-)	ATB (+)	*p* value
	n = 86	n = 19	
**Proteinuria, n (%)**	**38 (44.2)**	**7 (36.8)**	**0.558**
**Hypertension, n (%)**	**36 (41.9)**	**7 (36.8)**	**0.687**
**Fatigue, n (%)**	**30 (34.9)**	**3 (15.8)**	**0.105**
**Decreased appetite, n (%)**	**22 (25.6)**	**2 (10.5)**	**0.230**
**Fever, n (%)**	**13 (15.1)**	**3 (15.8)**	**1.000**
**Rash, n (%)**	**13 (15.1)**	**2 (10.5)**	**1.000**
**Increased AST or ALT, n (%)**	**12 (14.0)**	**3 (15.8)**	**0.733**
**Diarrhea, n (%)**	**8 (9.3)**	**4 (21.1)**	**0.223**
**Any infection, n (%)**	**7 (8.1)**	**1 (5.3)**	**1.000**
**Any adverse event of grade ≥ 3, n (%)**	**25 (29.1)**	**7 (36.8)**	**0.505**
**Any irAE treated with steroids, n (%)**	**5 (5.8)**	**1 (5.3)**	**1.000**

Abbreviations: AE, adverse event; ALT, alanine aminotransferase; AST, aspartate aminotransferase; ATB, antibiotics; irAE, immune-related adverse event.

In the ATB (-) group, a total of 37 patients discontinued the treatment. Of the 37 patients, 30 patients discontinued due to PD, and 7 patients discontinued due to the following AEs: fatigue, decreased appetite, decreased hepatic reserve, hepatic encephalopathy, spontaneous bacterial peritonitis, interstitial pneumonia, and gastrointestinal perforation. In the ATB (+) group, a total of 9 patients discontinued the treatment. Of the 9 patients, 7 patients discontinued due to PD, 1 patient discontinued due to pleurisy, and another as a result of cholangitis. The rates of discontinuation due to AEs did not differ between the ATB (-) and ATB (+) groups (8.1% vs. 10.2%; *p* = 0.665).

## Discussion

In this study, the percentage of radiological PD of the first therapeutic assessment was higher among patients with HCC who were administered ATB prior to atezolizumab plus bevacizumab than those in patients who were not administered ATB. This is the first study to report the effect of prior ATB use on the therapeutic responses of patients with HCC who were undergoing atezolizumab plus bevacizumab as the primary systemic therapy. Furthermore, the OS and PFS were significantly shorter in patients receiving prior ATB than those in patients not receiving prior ATB in this study. These results suggest an association between decreased therapeutic effect and prior ATB. However, differences in the backgrounds of patients receiving and not receiving prior ATB may have affected the differences in the patient prognoses between the groups, as prior ATB was not identified as an independent predictor associated with the OS and PFS in the multivariate analyses.

Previous studies have reported adverse effects of ATB use that affected therapeutic responses and prognosis in patients treated with ICIs for renal cell cancer, lung cancer, and melanoma [25–29]. Derosa et al [25]. reported that ATB use was associated with a 3.4-fold increase in the rate of primary PD in patients treated with ICIs for renal cell cancer. Cortellini et al [29]. reported that ATB use was correlated with a reduced probability of radiological response (odds ratio: 0.57, 95% confidence interval (CI): 0.37–0.87) and a high risk of mortality (hazard ratio (HR): 1.42, 95% CI: 1.13–1.79) in patients receiving pembrolizumab for non-small cell lung cancer. On the other hand, according to Hopkins et al [38]., antibiotic use was associated with worse survival in patients with urothelial carcinoma treated with atezolizumab. However, this association was not observed in patients treated with chemotherapy which suggests that ATB may specifically reduce the effectiveness of cancer immunotherapies. In patients with HCC, Cheung et al [30]. reported that ATB use during ICI therapy was associated with higher cancer-related (HR: 1.66, 95% CI: 1.08–2.54) and all-cause (HR: 1.63, 95% CI: 1.17–2.28) mortality. In contrast, Fessas et al [31]. reported an association between ATB exposure during ICI therapy HCC and prolonged PFS (HR: 0.75, 95% CI: 0.60–0.94) in patients with HCC. These inconsistent outcomes of ICI therapy associated with ATB use may be due to the varied treatment regimens such as nivolumab, pembrolizumab, and ipilimumab plus nivolumab used in the previous studies. The current study included patients receiving only atezolizumab plus bevacizumab based on the clinical practice guidelines, and the results of this study indicate that the prior use of ATB negatively affects the outcomes of this ICI therapy.

The definitions of the timing of exposure to ATB before or after ICI initiation vary from 30 to 60 days prior to ICI initiation in previous studies [25, 26, 28, 29]. Derosa et al [25]. reported that the impact of ATB used up to 60 days prior to ICI initiation was not as potent as within the first 30 days prior to ICI initiation. In other previous studies, the definitions of ATB use included the administration of ATB up to 30 days before and after ICI initiation [27, 30, 31]. Pinato et al [26]. reported that prior ATB use was associated with a worsened therapeutic response and poor prognosis in patients receiving ICI therapy, but that concurrent ATB use was not. In the present study, ATB use was defined as the administration of ATB up to 30 days prior to the atezolizumab plus bevacizumab initiation, and patients who were administered ATB within 30 days after the atezolizumab plus bevacizumab initiation were excluded from the analyses. To be precise, ATB should be used after diagnosing infection during ICI therapy but it was difficult to determine whether AEs were related to infection. Accordingly, all patients receiving ATB within 30 days after the atezolizumab plus bevacizumab initiation were excluded so that concurrent ATB use and early AEs requiring ATB use would not confound the treatment outcomes. Therefore, this study compares the treatment outcomes of patients receiving ATB prior to ICI therapy to those of patients who were not exposed to ATB up to 30 days before and after ICI initiation.

Marked diversity within and a high abundance of stool microbiota including *Akkermansia muciniphila*, *Ruminococcaceae*, and *Bifidobacterium longum* were associated with the therapeutic responses of patients with advanced cancer to ICI therapy [18–20]. Three bacterial species that enhanced the efficacy of ICIs (*Bifidobacterium pseudolongum*, *Lactobacillus johnsonii*, and *Olsenella* species) were isolated in mouse models [39]. Lee et al [40]. identified *Bifidobacterium pseudocatenulatum*, *Roseburia spp*, and *Akkermansia muciniphila*, which were associated with ICI responders, in patients with melanoma from five distinct cohorts, though no single bacterium was identified as a consistent biomarker across all cohorts. These results suggest that the human gut microbiome plays a complex role in ICI response. The biological mechanism of the effects of ATB on response to ICI therapy is unknown. In addition, Spencer et al [41]. reported that approximately 30% of patients with melanoma initiating ICI had taken sufficient dietary fiber intake and showed improved PFS compared with patients with insufficient dietary fiber intake. Prebiotics can affect the therapeutic responses of atezolizumab plus bevacizumab in patients receiving prior ATB, but dietary habits and intake of probiotic supplements were not considered in this study. Large stool sample studies including patients treated with ICIs are needed to elucidate the association between ICI response and the gut microbiome.

This study has some limitations. First, the retrospective design was limited by the short observation periods and the small sample size due to the recent approval of atezolizumab plus bevacizumab in patients with HCC. Second, the timing, duration, classes, doses, and purpose of ATB treatment with variable infection status and severity in the enrolled subjects were heterogeneous, and no subgroup analyses based on these variables were conducted. A few previous included such subgroup analyses. Most classes of ATB were insufficiently studied due to the small number of cases [28] and the difference in treatment duration of ATB had no effect on the results [30]. In addition, no microbiome analyses affected by these variables were conducted in the present study. Third, differences in patient background between the groups, such as patients with aggressive tumor burden/phenotype and poor immunity status probably included in the ATB (+) group, may have been a source of bias, though this bias was difficult to remove in clinical practice. In addition, patients with decompensated cirrhosis, which was likely to be complicated by the infection, could have potentially been included in the ATB (+) group.

In conclusion, prior ATB use was associated with reduced therapeutic responses to ICI therapy in patients with HCC undergoing atezolizumab plus bevacizumab as the primary systemic therapy.

## Supporting information

S1 Table Details regarding antibiotic treatment and duration in the ATB (+) group. (DOCX)

S2 Table Univariate and multivariate analyses of factors associated with PFS according to mRECIST. (DOCX)

S3 Table Univariate and multivariate analyses of factors associated with PFS according to RECIST v1.1. (DOCX)

S4 Table Univariate and multivariate analyses of factors associated with OS. (DOCX)

S1 Data Patient characteristics and data. (XLSX)

## Abbreviations

AE: adverse event
AFP: α-fetoprotein
ALBI: albumin-bilirubin
mALBI: modified ALBI
ALT: alanine aminotransferase
AST: aspartate aminotransferase
ATB: antibiotics
BCLC: Barcelona Clinic Liver Cancer
CI: confidence interval
CR: complete response
CRP: C-reactive protein
CT: computed tomography
DCR: disease control rate
ECOG PS: Eastern Cooperative Oncology Group performance status
ERCP: endoscopic retrograde cholangiopancreatography
EUS-FNA: endoscopic ultrasound-guided fine needle aspiration
HAIC: hepatic arterial infusion chemotherapy
HBV: hepatitis B virus
HCC: hepatocellular carcinoma
HCV: hepatitis C virus
HR: hazard ratio
ICI: immune checkpoint inhibitor
irAE: immune-related adverse event
MRI: magnetic resonance imaging
NLR: neutrophil-to-lymphocyte ratio
ORR: objective response rate
OS: overall survival
PD: progressive disease
PFS: progression-free survival
PR: partial response
RECIST v1.1: Response Evaluation Criteria in Solid Tumors version 1.1
mRECIST: modified RECIST
SD: stable disease
TACE: transarterial chemoembolization
WBC: white blood cell
