# Supplementary material for: Pretreatment with antibiotics is associated with reduced therapeutic response to atezolizumab plus bevacizumab in patients with hepatocellular carcinoma
Source: PLoS One. 2023 Feb 7;18(2):e0281459. doi: 10.1371/journal.pone.0281459 (PMC9904470; doi:10.1371/journal.pone.0281459)
Supplement: S1 Table — (DOCX) [file pone.0281459.s001.docx]

**S1 Table. Details regarding antibiotic treatment and duration in the ATB (+) group**

| **Patient** | **Antibiotics** | **Dose** | **Duration, days** | **Indications** | **Bacterial culture** | **Therapeutic responses of atezolizumab plus bevacizumab (mRECIST/RECIST v1.1)** |
| --- | --- | --- | --- | --- | --- | --- |
| **1** | **Cefmetazole** | **1g, q12h** | **16** | **After TACE** | **None** | **PD/PD** |
| **2** | **Cefmetazole** | **1g, q12h** | **15** | **Hepatic abscess** | ***Klebsiella pneumoniae*** | **SD/SD** |
| **3** | **Cefmetazole** | **1g, q12h** | **12** | **Cholecystitis** | **Negative** | **NE/NE** |
| **4** | **Cefmetazole** | **1g, q12h** | **2** | **After TACE** | **None** | **SD/SD** |
| **5** | **Sulbactam/Cefoperazone** | **1g, q12h** | **11** | **After HAIC** | **None** | **PR/SD** |
| **6** | **Sulbactam/Cefoperazone** | **1g, q12h** | **8** | **After TACE** | **None** | **PD/PD** |
| **7** | **Sulbactam/Cefoperazone** | **1g, q12h** | **2** | **After EUS-FNA** | **None** | **PR/PR** |
| **8** | **Sulbactam/Cefoperazone** | **1g, q24h** | **2** | **After EUS-FNA** | **None** | **SD/SD** |
| **9** | **Cefazolin** | **1g, q24h** | **1** | **After liver biopsy** | **None** | **PR/SD** |
| **10** | **Cefazolin** | **1g, q24h** | **1** | **After liver biopsy** | **None** | **PR/SD** |
| **11** | **Cefepime** | **1g, q12h** | **13** | **Cholecystitis** | ***Citrobacter braakii*** | **PD/PD** |
| **12** | **Cefpodoxime Proxetil** | **100mg, twice a day** | **5** | **Cystitis** | **None** | **PD/PD** |
| **13** | **Sulbactam/Ampicillin** | **1.5g, q8h** | **14** | **Cholangitis** | ***Enterobacter aerogenes*** | **PD/PD** |
| **14** | **Tazobactam/Piperacillin** | **4.5g, q8h** | **5** | **After ERCP** | **None** | **PD/PD** |
| **15** | **Amoxicillin/Clavulanate** | **Amoxicillin 250mg/Clavulanate 125mg, 3 times a day** | **23** | **Pyothorax** | **Negative** | **PR/PR** |
| **16** | **Levofloxacin** | **500mg, once a day** | **5** | **Cholangitis** | **Negative** | **PD/PD** |
| **17** | **Teicoplanin** | **400mg, q24h** | **14** | **Hepatic abscess** | ***Enterococcus faecium*** | **SD/PD** |
| **18** | **Rifaximin** | **400mg, 3 times a day** | **> 30** | **Hepatic encephalopathy** | **None** | **PD/PD** |
| **19** | **Rifaximin** | **400mg, 3 times a day** | **> 30** | **Hepatic encephalopathy** | **None** | **PD/PD** |
| **Abbreviations: ATB, antibiotics;** **ERCP, endoscopic retrograde cholangiopancreatography; EUS-FNA, endoscopic ultrasound-guided fine needle aspiration;** **HAIC, hepatic arterial infusion chemotherapy; mRECIST, modified Response Evaluation Criteria in Solid Tumors; PD, progressive disease; PR, partial response; RECIST v1.1, Response Evaluation Criteria in Solid Tumors version 1.1; SD, stable disease;** **TACE, transarterial chemoembolization.** | | | | | | |
