# Supplementary material for: Pretreatment with antibiotics is associated with reduced therapeutic response to atezolizumab plus bevacizumab in patients with hepatocellular carcinoma
Source: PLoS One. 2023 Feb 7;18(2):e0281459. doi: 10.1371/journal.pone.0281459 (PMC9904470; doi:10.1371/journal.pone.0281459)
Supplement: S2 Table — (DOCX) [file pone.0281459.s002.docx]

**S2 Table. Univariate and multivariate analyses of factors associated with PFS according to mRECIST**

| **Variable** | **Category** | **Univariate analysis** | ***p* value** | **Multivariate analysis** | ***p* value** |
| --- | --- | --- | --- | --- | --- |
|  |  | **Hazard ratio (95% CI)** |  | **Hazard ratio (95% CI)** |  |
| **Age, years** | **≥ 75** | **0.704 (0.396-1.252)** | **0.232** |  |  |
| **Sex** | **Female** | **0.981 (0.487-1.978)** | **0.958** |  |  |
| **ECOG PS** | **1** | **1.030 (0.319-3.324)** | **0.960** |  |  |
| **Etiology** | **Non-viral** | **0.948 (0.738-1.148)** | **0.585** |  |  |
| **Child-Pugh score** | **6 or 7** | **1.313 (0.739-2.334)** | **0.353** |  |  |
| **mALBI grade** | **2b or 3** | **11.723 (0.969-3.064)** | **0.064** |  |  |
| **Platelet count, x 10^4^/μL** | **≤ 14.0** | **1.500 (0.837-2.688)** | **0.173** |  |  |
| **Maximum intrahepatic tumor size, mm** | **≥ 50** | **1.586 (0.855-2.942)** | **0.143** |  |  |
| **Intrahepatic tumor number** | **≥ 5** | **1.998 (1.124-3.552)** | **0.018** | **2.064 (1.155-3.688)** | **0.014** |
| **Macrovascular invasion** | **Present** | **3. 606 (1.922-6.764)** | **< 0.001** | **2.953 (1.531-5.696)** | **0.001** |
| **Extrahepatic metastasis** | **Present** | **1.397 (0.781-2.500)** | **0.260** |  |  |
| **BCLC stage** | **C** | **2.252 (1.222-4.150)** | **0.009** |  |  |
| **AFP, ng/mL** | **≥ 400** | **1.628 (0.853-3.108)** | **0.139** |  |  |
| **NLR** | **≥ 3** | **1.956 (1.078-3.549)** | **0.027** | **1.790 (0.961-3.331)** | **0.066** |
| **CRP, mg/dL** | **≥ 0.25** | **1.919 (1.067-3.454)** | **0.030** | **1.642 (0.864-3.121)** | **0.130** |
| **ATB** | **With** | **2.036 (1.033-4.016)** | **0.040** | **1.413 (0.661-3.018)** | **0.372** |
| **Abbreviations: AFP, α-fetoprotein; ATB, antibiotics; BCLC, Barcelona Clinic Liver Cancer; CI, confidence interval; CRP, C-reactive protein; ECOG PS, Eastern Cooperative Oncology Group performance status; mALBI, modified albumin-bilirubin; mRECIST, modified Response Evaluation Criteria in Solid Tumors; NLR, neutrophil-to-lymphocyte ratio; PFS, progression-free survival.** | | | | | |
