# Supplementary material for: Pretreatment with antibiotics is associated with reduced therapeutic response to atezolizumab plus bevacizumab in patients with hepatocellular carcinoma
Source: PLoS One. 2023 Feb 7;18(2):e0281459. doi: 10.1371/journal.pone.0281459 (PMC9904470; doi:10.1371/journal.pone.0281459)
Supplement: S3 Table — (DOCX) [file pone.0281459.s003.docx]

**S3 Table. Univariate and multivariate analyses of factors associated with PFS according to RECIST v1.1**

| **Variable** | **Category** | **Univariate analysis** | ***p* value** | **Multivariate analysis** | ***p* value** |
| --- | --- | --- | --- | --- | --- |
|  |  | **Hazard ratio (95% CI)** |  | **Hazard ratio (95% CI)** |  |
| **Age, years** | **≥ 75** | **0.724 (0.409-1.281)** | **0.267** |  |  |
| **Sex** | **Female** | **0.916 (0.455-1.845)** | **0.807** |  |  |
| **ECOG PS** | **1** | **0.989 (0.307-3.189)** | **0.986** |  |  |
| **Etiology** | **Non-viral** | **0.972 (0.804-1.175)** | **0.769** |  |  |
| **Child-Pugh score** | **6 or 7** | **1.248 (0.707-2.203)** | **0.444** |  |  |
| **mALBI grade** | **12b or 3** | **1.698 (0.962-2.997)** | **0.068** |  |  |
| **Platelet count, x 10^4^/μL** | **≤ 14.0** | **1.596 (0.894-2.849)** | **0.114** |  |  |
| **Maximum intrahepatic tumor size, mm** | **≥ 50** | **1.684 (0.917-3.094)** | **0.093** |  |  |
| **Intrahepatic tumor number** | **≥ 5** | **1.841 (1.043-3.251)** | **0.035** | **1.898 (1.062-3.393)** | **0.031** |
| **Macrovascular invasion** | **Present** | **3. 450 (1.852-6.430)** | **< 0.001** | **2.903 (1.511-5.578)** | **0.001** |
| **Extrahepatic metastasis** | **Present** | **1.539 (0.863-2.744)** | **0.144** |  |  |
| **BCLC stage** | **C** | **2.444 (1.325-4.507)** | **0.004** |  |  |
| **AFP, ng/mL** | **≥ 400** | **1.555 (0.817-2.962)** | **0.179** |  |  |
| **NLR** | **≥ 3** | **1.902 (1.052-3.439)** | **0.033** | **1.770 (0.955-3.280)** | **0.070** |
| **CRP, mg/dL** | **≥ 0.25** | **1.800 (1.009-3.210)** | **0.046** | **1.650 (0.869-3.134)** | **0.126** |
| **ATB** | **With** | **1.946 (0.988-3.831)** | **0.054** | **1.351 (0.631-2.894)** | **0.439** |
| **Abbreviations: AFP, α-fetoprotein; ATB, antibiotics; BCLC, Barcelona Clinic Liver Cancer; CI, confidence interval; CRP, C-reactive protein; ECOG PS, Eastern Cooperative Oncology Group performance status; mALBI, modified albumin-bilirubin; NLR, neutrophil-to-lymphocyte ratio; PFS, progression-free survival; RECIST v1.1, Response Evaluation Criteria in Solid Tumors version 1.1.** | | | | | |
