# Supplementary material for: Pretreatment with antibiotics is associated with reduced therapeutic response to atezolizumab plus bevacizumab in patients with hepatocellular carcinoma
Source: PLoS One. 2023 Feb 7;18(2):e0281459. doi: 10.1371/journal.pone.0281459 (PMC9904470; doi:10.1371/journal.pone.0281459)
Supplement: S4 Table — (DOCX) [file pone.0281459.s004.docx]

**S4 Table. Univariate and multivariate analyses of factors associated with OS**

| **Variable** | **Category** | **Univariate analysis** | ***p* value** | **Multivariate analysis** | ***p* value** |
| --- | --- | --- | --- | --- | --- |
|  |  | **Hazard ratio (95% CI)** |  | **Hazard ratio (95% CI)** |  |
| **Age, years** | **≥ 75** | **1.258 (0.446-3.544)** | **0.664** |  |  |
| **Sex** | **Female** | **1.907 (0.651-5.587)** | **0.239** |  |  |
| **ECOG PS** | **1** | **0.979 (0.128-7.472)** | **0.984** |  |  |
| **Etiology** | **Non-viral** | **0.785 (0.548-1.123)** | **0.185** |  |  |
| **Child-Pugh score** | **6 or 7** | **7.167 (1.615-31.802)** | **0.010** | **12.329 (2.218-68.523)** | **0.004** |
| **mALBI grade** | **2b or 3** | **3.574 (1.136-11.247)** | **0.029** |  |  |
| **Platelet count, x 10^4^/μL** | **≤ 14.0** | **1.610 (0.549-4.719)** | **0.386** |  |  |
| **Maximum intrahepatic tumor size, mm** | **≥ 50** | **3.805 (1.334-10.855)** | **0.012** | **0.931 (0.229-3.785)** | **0.921** |
| **Intrahepatic tumor number** | **≥ 5** | **3.029 (1.033-8.881)** | **0.043** | **5.222 (1.439-18.944)** | **0.012** |
| **Macrovascular invasion** | **Present** | **5.048 (1.733-14.706)** | **0.003** | **6.383 (1.519-26.827)** | **0.011** |
| **Extrahepatic metastasis** | **Present** | **1.461 (0.528-4.038)** | **0.465** |  |  |
| **BCLC stage** | **C** | **2.958 (0.939-9.319)** | **0.064** |  |  |
| **AFP, ng/mL** | **≥ 400** | **4.399 (1.529-12.656)** | **0.006** | **3.453 (1.071-11.132)** | **0.038** |
| **NLR** | **≥ 3** | **2.828 (1.014-7.886)** | **0.047** | **3.480 (1.061-11.411)** | **0.040** |
| **CRP, mg/dL** | **≥ 0.25** | **5.553 (1.560-19.762)** | **0.008** | **3.622 (0.885-14.814)** | **0.073** |
| **ATB** | **With** | **3.581 (1.199-10.696)** | **0.022** | **1.202 (0.293-4.933)** | **0.798** |
| **Abbreviations: AFP, α-fetoprotein; ATB, antibiotics; BCLC, Barcelona Clinic Liver Cancer; CI, confidence interval; CRP, C-reactive protein; ECOG PS, Eastern Cooperative Oncology Group performance status; mALBI, modified albumin-bilirubin; NLR, neutrophil-to-lymphocyte ratio; OS, overall survival; RECIST v1.1, Response Evaluation Criteria in Solid Tumors version 1.1.** | | | | | |
